# Supplementary material for: Splice-Junction-Based Mapping of Alternative Isoforms in the Human Proteome
Source: Cell Rep. Author manuscript; Available in PMC 2020 Jan 15. (PMC6961840; doi:10.1016/j.celrep.2019.11.026)
Supplement: 2 [file NIHMS1546469-supplement-2.zip › DF1/PXD010154/Lung/Lung_16_TNXB_MGPLSVVIVTVAR.pdf]

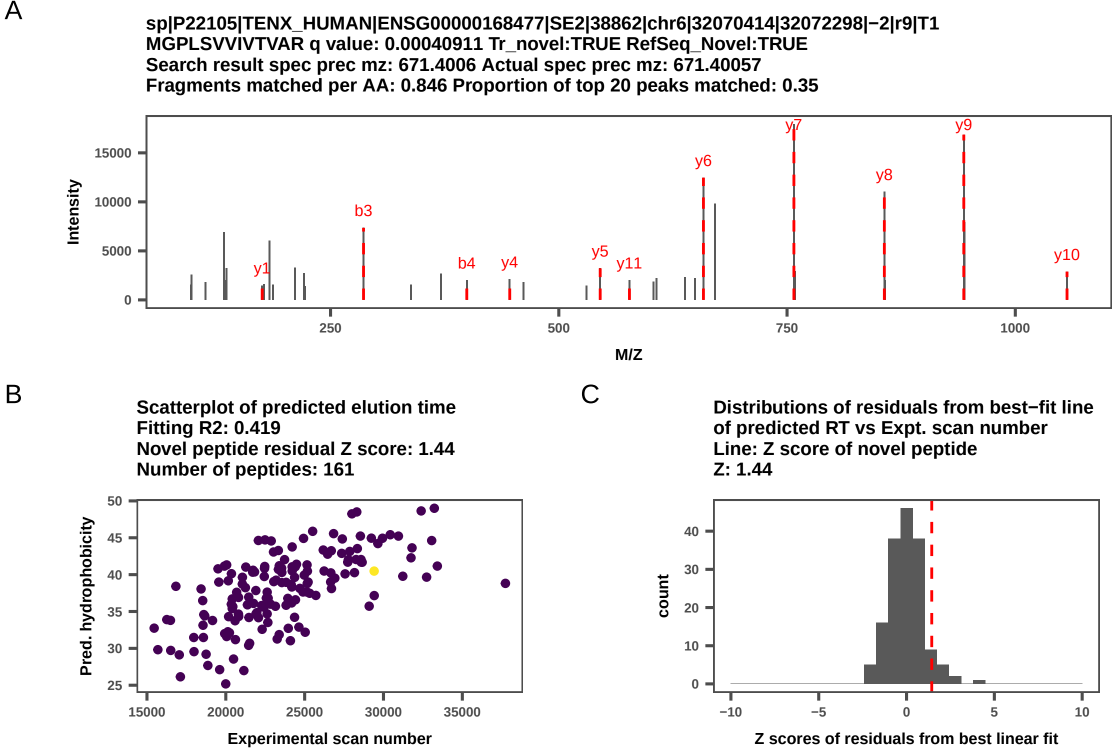
A
sp|P22105|TENX_HUMAN|ENSG00000168477|SE2|38862|chr6|32070414|32072298|−2|r9|T1
MGPLSVVIVTVAR q value: 0.00040911 Tr_novel:TRUE RefSeq_Novel:TRUE
Search result spec prec mz: 671.4006 Actual spec prec mz: 671.40057
Fragments matched per AA: 0.846 Proportion of top 20 peaks matched: 0.35
Intensity
M/Z
y1
b3
b4
y4
y5
y11
y6
y7
y8
y9
y10
B
Scatterplot of predicted elution time
Fitting R2: 0.419
Novel peptide residual Z score: 1.44
Number of peptides: 161
Pred. hydrophobicity
Experimental scan number
C
Distributions of residuals from best−fit line
of predicted RT vs Expt. scan number
Line: Z score of novel peptide
Z: 1.44
count
Z scores of residuals from best linear fit
